# Assessing the Welfare of Captive Group-Housed Cockroaches, *Gromphadorhina oblongonota*

**DOI:** 10.3390/ani13213351

**Published:** 2023-10-27

**Authors:** Danielle Free, Sarah Wolfensohn

**Affiliations:** 1 Marwell Wildlife, Winchester SO21 1JH, UK; danif@marwell.org.uk; 2 School of Veterinary Medicine, University of Surrey, Guildford GU2 7AL, UK

**Keywords:** Animal Welfare Assessment Grid (AWAG), group welfare, invertebrate, sentience, cockroach, quality of life, welfare

## Abstract

**Simple Summary:**

Invertebrate welfare is gaining attention, especially with the rise of insect farming for sustainable food production. Traditional individual welfare monitoring is impractical for large groups, especially when individual identification is difficult. This study adapts the Animal Welfare Assessment Grid (AWAG) for group-level assessments and successfully applies it to a captive group of male *Gromphadorhina oblongonota*. This modified AWAG evaluates welfare based on 12 factors tracked over time, revealing environmental and social factors’ impact on *G. oblongonota* welfare. These findings guide practical improvements in care and offer an efficient method to assess invertebrate welfare at the group level.

**Abstract:**

The welfare of invertebrates under human care is of growing concern, particularly with the increasing interest in insect farming as an environmentally sustainable means of producing food. Additionally, individual welfare monitoring systems can be time-consuming and impractical for larger groups, particularly when individual animals are difficult to identify. It is, therefore, imperative to develop a validated system for monitoring terrestrial invertebrate welfare at a group level. The Animal Welfare Assessment Grid (AWAG) is an objective welfare-monitoring tool that has been approved for use with a wide range of species. This study modified the AWAG for large group-level welfare assessments and successfully trialled it on a terrestrial invertebrate species, a group of captive male *Gromphadorhina oblongonota*. The modified template evaluated the group’s welfare by scoring changes to 12 factors that could be tracked over time. The results highlight that the welfare of *G. oblongonota* is likely to be influenced by environmental and social factors, and inform practical improvements in *G. oblongonota* care that will result in improved welfare. The findings also demonstrate an efficient way to assess the welfare of invertebrates at the group level, and given the recent UK legislation (Animal Welfare (Sentience) Bill, 2022) plus the emerging interest in invertebrate farming, our findings hold timely significance.

## 1. Introduction

The welfare of invertebrates under human care is of growing concern [1,2,3,4]. Invertebrates are extensively used in laboratories, as ‘live food’ (to feed non-human animals, e.g., pets, zoo animals and livestock), as pets, for educational purposes (e.g., in classrooms, zoos and museums), and for pest control. There is also increasing interest in farming invertebrates for human consumption (human entomophagy) [5]. There are no definitive figures for the number of invertebrates currently in captivity in the UK, and what data are available typically refer to invertebrates by weight (e.g., kilograms or tonnes), but they likely number in the billions. For example, in 2021, 6000 tonnes of UK-reared insect meal were used in feed for fish farms, pigs and poultry alone. The World Wildlife Foundation (WWF) predict that, by 2050, UK-reared insect meal could be increased to 237,000 tonnes [6]. With such high numbers of invertebrates involved, the importance of assessing invertebrate welfare cannot be underestimated.

Unlike vertebrates, invertebrates receive little protection under UK law. Following the UK’s withdrawal from the European Union, Article 13 of the Treaty on the Functioning of the European Union (TFEU), which stated that animals are sentient beings and conferred a duty on member states to pay full regard to animal welfare when formulating and implementing policy [7], was not carried over into UK law. Motivated by public concern and moral consideration for animal welfare, the UK Government passed the Animal Welfare (Sentience) Bill in 2022. Following the provision of scientific evidence of sentience, some invertebrates (e.g., octopuses and lobsters [8]), were included in the bill; however, sentience for the majority of invertebrate species is still not recognised. ‘Sentience’ is defined by the Farm Animal Welfare Council (FAWC), 2018, as: ‘*…the capability to experience pain, distress and harm*’ [9]. Although scientists are still unable to prove sentience for most invertebrate species [10], many agree that, as sentience cannot be disproved, the precautionary principle should be employed; that is, invertebrates should be given the benefit of the doubt with regard to their ability to suffer [1,3,11,12,13].

Given the lack of legislation, there is a lack of guidance on how to care for invertebrates in captivity. Most available guidance focusses on abiotic conditions. For example, most invertebrates are ectothermic and maintaining a suitable temperature for these species in captivity is key. Lack of guidance also stems from a deficiency of behavioural and ecological data, such as species-specific reproductive and feeding behaviours. This paucity of data is exacerbated by the vast number and diversity of invertebrate species, resulting in ‘trial and error’ husbandry [4,14], which, if successful for survival and breeding, can result in folklore husbandry, where husbandry methods persist because they have always been used [15].

These hurdles have led to the welfare of invertebrates often being overlooked by society. The World Association of Zoos and Aquariums (WAZA) defines animal welfare as ‘*…a state that is specific for every individual animal; it is how the animal experiences its own world and life through its association with pleasant experiences specific for that species…or unpleasant experiences*’ [16]. There are, however, many definitions of the term ‘welfare’, and growing arguments there is no single definition that is all-encompassing [17], with some arguing that the term can only apply to sentient living organisms [18,19]. Although welfare indicators for invertebrates in general are lacking, welfare has been well-studied for vertebrates, from which we can generalise that certain elements of captivity could negatively impact invertebrate welfare. Moreover, as more is understood about sentience in invertebrates and their ability to feel pain, it may be advantageous to employ the precautionary principle and afford invertebrates the benefit of the doubt until confirmed otherwise. Following the precautionary principle, however, and considering the colossal number of individuals at risk, it is important that industries involving living invertebrates identify methods for assessing their welfare [13].

In the UK, legislation discourages the feeding of live vertebrate prey to predators (Animal Welfare Act 2006 and Zoo Licensing Act 1981); however, this legislation is not extended to invertebrate species, with zoos both buying in and breeding invertebrates on site for ‘feeding out’. The feeding of live invertebrate prey is believed to be enriching and beneficial for the welfare of the predator, with the welfare of the invertebrates only recently becoming a concern [20,21,22]. Invertebrates in zoos, particularly those bred for feeding out, are often managed in large numbers in comparatively small enclosures (personal experience). Due to the need to assess multiple individuals and time intervals, assessing the welfare of large groups can be time-consuming, costly and impractical [23,24]. It is especially difficult to individually assess species that cannot be easily identified at an individual level and this is further compounded by the use of larger and more complex environments to house animals in zoos and aquariums [24], highlighting the need for a practical and valid group-level welfare assessment tool.

This exploratory study adapts the Animal Welfare Assessment Grid (AWAG) designed by Narshi et al. [25] to assess its efficacy as a group-level welfare monitoring tool for terrestrial invertebrate species. The AWAG is a practical online cloud-based software [26] devised to assess and monitor the welfare and cumulative lifetime experience of animals. The AWAG tool encompasses the five domains of welfare (see Mellor et al. [27]) across the following four parameters:

*Physical:* assesses an animal’s clinical health, including factors such as body condition, illness and injury.

*Behavioural/Psychological:* assesses an animal’s mental wellbeing and includes factors such as behavioural response to stressors and how often these are encountered. Animals cannot verbally communicate their emotions; therefore, behaviour is used as an indicator to explore their psychological health.

*Environmental:* assesses the animal’s environment, whether it is both suitable and complex, options for social opportunities, and with choice and comfort.

*Procedural:* assesses how the animal responds to clinical and husbandry events, and includes factors such as handling, changes in routine, and pain from veterinary or management procedures.

The AWAG is unique in that it considers the lifetime experience of the animal, and the cumulative suffering that can impact quality of life. The tool provides a mean score for factors in each parameter and plots these on a grid to create a minimum convex polygon, the area of which is the cumulative welfare assessment score (CWAS) for that moment in time. The CWAS can then be tracked across the animal’s lifetime to assess quality of life, allowing for the user to quantify welfare and assess whether treatment or changes in management systems are required or have been successful in improving welfare. The AWAG allows for the user to drill down and identify which factors are positively or negatively affecting welfare and make focused interventions. This tool has been used for a variety of species and various environments, including zoos, farms, companion animal care, and research laboratories [25,28,29,30,31,32]; however, use with large groups of terrestrial invertebrates has yet to be trialled.

## 2. Materials and Methods

This study modified the AWAG designed by Narshi et al. [25] for assessing the welfare of decapod and cephalopod invertebrates for use with a captive group of *Gromphadorhina oblongonota*, as a proxy for large groups of terrestrial invertebrates housed in captivity. We reviewed the scientific literature for validated welfare indicators for the species on which to base the welfare assessment; however, the literature specific to *G. oblongonota* was scarce, and with over 4000 species of cockroach inhabiting vastly different environments worldwide, extrapolating information from some of the better-understood species, including those regarded as domestic pests or those used in laboratories, was difficult. Where information was available, it largely concerned eradication methods, social behaviour, allergens, neurophysiology, and endocrinology, not welfare indicators.

As per Free et al. [33], we compensated for the lack of peer-reviewed literature by gathering information from commercial and hobbyist communities (via communication with experts through social media and e-mail, and reviewing associated websites, forums, and social media groups), other species within the genus, and direct behavioural observations.

### 2.1. Study Subjects

**Figure 1:**
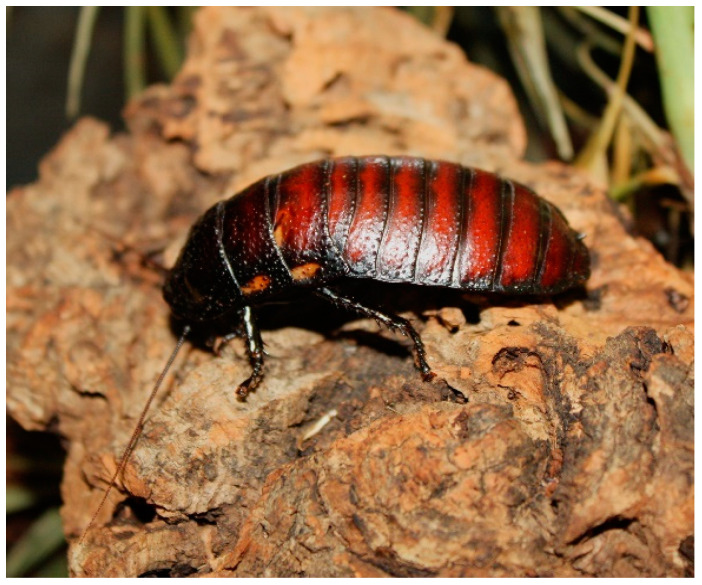
*Gromphadorhina oblongonota* (Marwell Wildlife).

**Figure 2:**
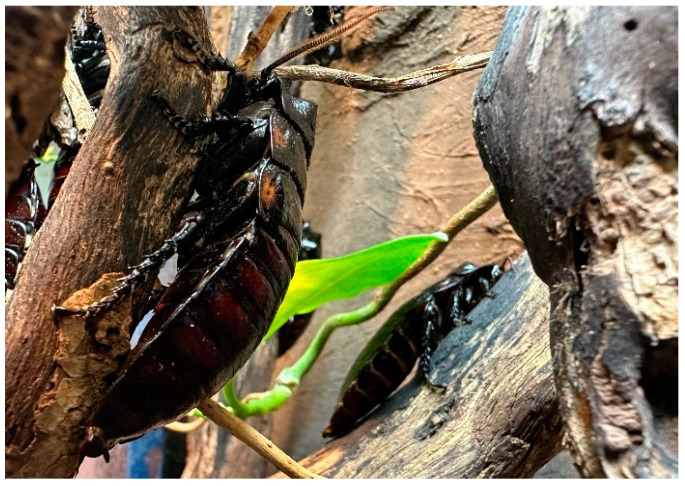
*Gromphadorhina oblongonota* (Kilroy, D).

The subjects of this study were a zoo-housed group of 31 male *Gromphadorhina oblongonota* at the zoological institution Marwell Zoo (UK). Species of the genus *Gromphadorhina* are managed by many zoos as food for omnivorous and insectivorous species, as well as for educational purposes. The group monitored in this study were housed on-show for viewing by zoo guests. *G. oblongonota* (Figure 1 and Figure 2)*,* also known as the wide-horned hisser, are one of the largest cockroach species in the world, measuring between 5 cm and 10 cm (adult), and are found in southern Madagascar. They are flightless and produce a hissing sound by expelling air through modified spiracles [34,35,36].

**Figure 3:**
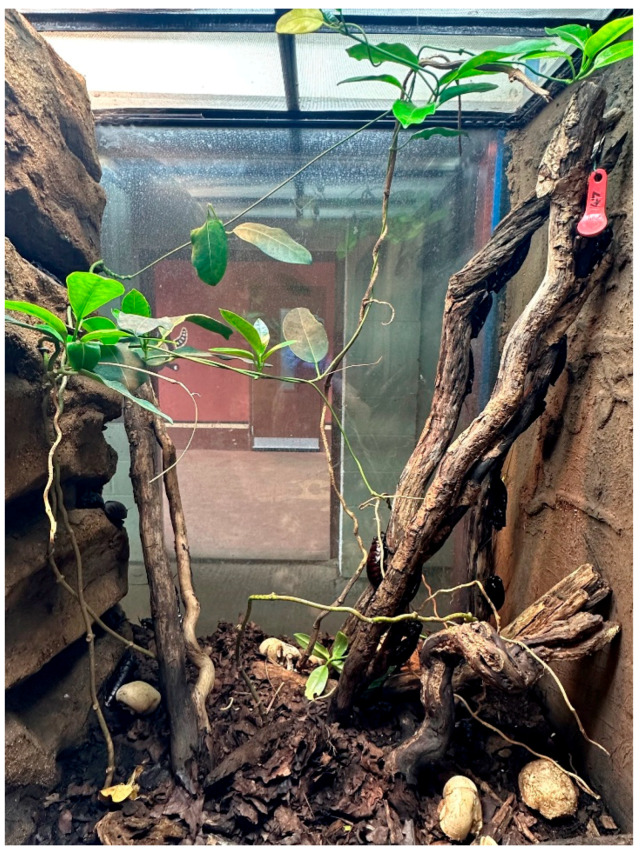
*Gromphadorhina oblongonota* enclosure, Marwell Zoo (Kilroy, D).

The group is all-male to prevent overpopulation and is supplemented with males removed from a breeding population used at the zoo for feeding-in. We chose this group to eliminate the need to assess welfare for different life stages and sexes, which have different environmental requirements and a different diet to adult males [35,37,38]. The group were housed in a glass vivarium (Figure 3) measuring 60 × 45 × 90 cm (L × D × H) with a secure mesh upper panel and hinged door for the front. The mesh upper panel allowed for ventilation, lighting (12% Arcadia T5, 6500 K plant light on a seasonal 13:11–11:13 cycle), some UV (low level < 2.0 UVI) and heating (overhead flood halogen 120 W, plus indirect heat from UV/plant lighting). The enclosure had a deep layer of substrate with regular additions of natural leaflitter, maintained by a clean-up-crew of isopods, so additional cleaning was not required. Branching and natural-looking rock facades on two sides of the enclosure completed the environment. The enclosure was misted twice daily to increase the humidity and provide drinking water. Food was typically replaced twice a week and consisted of seasonal browse, fungi and some produce. The *G. oblongonota* were only handled when being added to the enclosure (after removal from the separate breeding colony) or once deceased.

### 2.2. Experimental Design

We assessed the factors utilised by Narshi et al. [25] for their relevance for *Gromphadorhina oblongonota* and ability to be scored accurately at the species and group level, removing those that were not suitable. Following this process, we adapted the remaining scoring definitions by combining elements from previously tested group AWAGs for other species [29] and species-specific criteria for *G. oblongonota*. We detail the final factors below.

**Physical** (Table 1): The physical parameter considered three animal/outcome-based factors: general condition, presence of injury and activity level. ‘General condition’ assessed the appearance of the carapace (dull/shiny), body morphology (with both shrivelled and swollen abdomens a concern), difficulty moulting and density of mites [39]. *Gromphadorhina* spp. act as hosts for the mite *Androlaelaps schaeferi* (previously named *Gromphadorholaelaps schaeferi*), and are commensal, feeding on the same food as their host rather than the host itself; therefore, they are not of concern [39,40,41]. As *A. schaeferi* are primarily found in groups around the spiracles and between the legs of the cockroach [40], they are easy to identify. We merged ‘Presence of injury’ and ‘Observable clinical signs’ from Narshi et al. [25], as there is insufficient information in the literature on recognised clinical symptoms for *G. oblongonota*. ‘Activity level’ simply looked at the percentage of individuals that showed signs of activity during the observation period: 9.30 a.m.–4.30 p.m. We considered activity during the day an indicator of reduced welfare due to the impact on circadian rhythm [42] and the evidence that sleep deprivation in cockroaches and other invertebrates impacts memory formation [43] and can lead to increased metabolic rate, with severe sleep deprivation resulting in death [44]. ‘Food intake’ was removed, as the amount and type of items fed were too variable to determine accurate changes in the quantity consumed (there was always a large amount remaining when fed again), plus, as food was available 24/7 and *G. oblongonota* are nocturnal [36,45,46], they were rarely seen feeding.

**Psychological** (Table 2): The psychological parameter measured three factors: abnormal behaviour, response to guest presence and social interaction. ‘Abnormal behaviour’ considered excessive hissing [38] and increased speed of movement. We changed ‘Response to social disruption’ to ‘Response to guest presence’, firstly due to the little interaction the keepers had with the cockroaches, and secondly as the tank was situated in an area of the zoo that can become busy and noisy with guests, who can approach close to the tank. ‘Social interaction’ was added as an additional factor due to the abundance of the published literature relating to the hierarchal and aggressive behaviour exhibited by male *Gromphadorhina* spp. [47,48,49,50]. ‘Routine management’ from Narshi et al. [25] was removed, as little routine husbandry, other than misting and food input/removal, was carried out, and these were scored under the procedural parameter instead.

**Environmental** (Table 3): The environmental parameter measured five factors: environment, group size and structure, enclosure complexity, nutrition, and contingent events. Most of these factors are resource/input-based, analysing what resources the cockroaches have been provided or what they can access, as well as the subsequent effects of these on the cockroaches’ overall welfare. Although resource/input-based factors do not account for whether the animals use the provided resources, the lack of validated welfare indicators for invertebrates required us to utilise all means that were available [33]. The factor ‘Environment’ considered various environmental elements, for example, light, light cycle, UV and cleanliness. Due to their being ectotherms, the temperature, humidity and ventilation in the enclosure was of particular concern [14,35], but ambient noise level and guest proximity were also considered. ‘Group size and structure’ was essential to assess since overcrowding can lead to disturbances in circadian rhythm, a greater frequency of aggression, reduced growth rates and higher mortality [37,42]. Due to the inability to observe the cockroaches when they were most active, and thus be able to assess the quality and quantity of the natural behaviour that was exhibited, we evaluated ‘Enclosure complexity’, which focussed on the complexity of the enclosure features and layout, e.g., branching and substrate, and the behaviours that these features enabled the cockroaches to exhibit, e.g., foraging, burrowing, and successful moulting, instead. ‘Nutrition’ assessed the components of the diet in relation to wild and captive diets and considered that diet presentation likely resulted in feeding and foraging behaviours. Finally, ‘Contingent events’ considered the impact of irregular events, e.g., the addition of new individuals to the enclosure, guest events or nearby building work, on welfare. For the most part, these scores remained constant as the environment did not change throughout the study. We did not include ‘Water quality’ and ‘Accessibility’ from Narshi et al. [25], as neither were relevant.

**Procedural** (Table 4): The procedural parameter evaluated one factor: the effect of intervention. We removed ‘Isolation/Restraint’, ‘Impact of veterinary procedures’, ‘Change in daily routine’ and ‘Sedation/Anaesthesia’ from Narshi et al. [25], as veterinary interventions were very unlikely to occur for this group and daily routine was variable anyway.

We modified many of the physical, psychological and procedural parameter factors to adapt the AWAG template designed by Narshi et al. [25] for group-level assessment. For six of the factors, this involved using a percentage of the group for each definition, from 1 to 10. In most cases, a score of 1 indicated that the whole group had ideal welfare for that factor, decreasing by 1–10% for scores 2–9, and a score of 10 indicated that more than 80% of the group had the poorest welfare for that factor. Using a percentage range when scoring allowed for those individuals that could not be seen at each observation.

‘Social behaviour’ included a mixture of number of aggressive interactions, severity of interaction and the percentage of the group involved in interactions. The five environmental factors did not require adaptation to remain relevant for a group.

The final AWAG consisted of 12 factors within the four parameters, with each scored incrementally on a scale from 1 (best welfare state) to 10 (worst welfare state). We carefully defined each score to enhance interscorer agreement and, during preliminary observations, baseline scores were collectively agreed on by two independent observers, who also scored the group’s welfare simultaneously on two occasions during these observations. Interscorer agreement was then determined by calculating the percentage of scores that varied between the two scorers and was calculated at 100%. The observers were undergraduate students of relevant disciplines that were trained by the primary author. See Justice et al. [29] and Brouwers and Duchateau [51] for further detail on the methods used. The score definitions are presented in Table 1, Table 2, Table 3 and Table 4.

### 2.3. Welfare Analysis

Discussions with the keeping team and four preliminary observations across two days allowed a baseline score for the group to be established. We counted injuries during this period, as each individual was seen at least once. Five injuries were identified and fed into the baseline score, which only changed if additional injuries were noted, or an injured individual died. As it was not always possible to see all the individuals at each observation without disruption which would negatively impact welfare, those out of sight were scored at the baseline level.

Two or three welfare assessments were conducted per day between 09:30 and 16:30, between 11 and 21 September 2023. Multiple assessments were conducted per day to detect differences in the group’s welfare over the course of a day as they experienced changes in temperature, humidity, presence/absence of food, guest numbers etc. Multiple assessments also increased the ability to see all individuals. The group was monitored for 15 min per observation and scored using the modified AWAG. The scores were input into the AWAG software (https://awag.org.uk/, accessed on 23 September 2023) [26] for analysis. The AWAG cloud-based software generates an average for each parameter and plots this as a minimum convex polygon graph for every welfare assessment. The Cumulative Welfare Assessment Score (CWAS) is derived from the area of the polygon [25,29,31]. Temperature in the tank was recorded using a DS1921G-F5 Thermochron^®^ iButton^®^ [52], which noted the temperature in degrees Celsius every 2 h.

The study received the University of Surrey’s and Marwell Wildlife’s ethical approval prior to data collection.

## 3. Results

The total number of individuals in the enclosure was 31 at the start of the data collection period and 30 at the end due to the death of one individual from natural causes.

**Figure 4:**
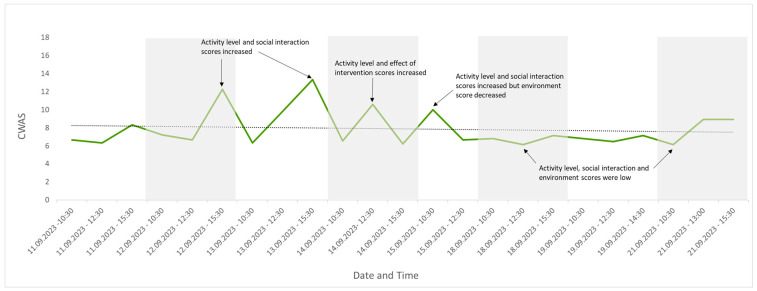
The CWAS for the group over the study period. The lower the score, the better the welfare. This form of visual representation highlights specific events that may be impacting welfare, in addition to changes in the trend of welfare over time, with a rising line indicating welfare is declining or a falling line indicating welfare is improving. Annotations identify which factor scores changed as a result of specific events, indicating a decline (peak) or improvement (trough) in group welfare.

**Figure 5:**
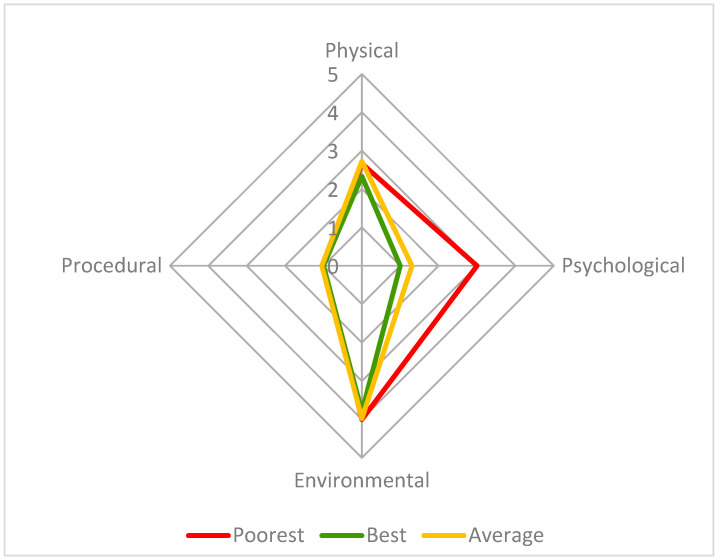
This radar chart compares the four parameter scores (physical, psychological, environmental, and procedural) for three welfare assessments: 13 September 15:30 (poorest group welfare score—13.3, red), 21 September 10:30 (best group welfare score—6.1, green) and the average for the study period (7.9, orange), on a scale from 1 to 10, with 1 being the best possible score and 10 the poorest. The axes in this figure are adjusted to improve readability.

**Figure 6:**
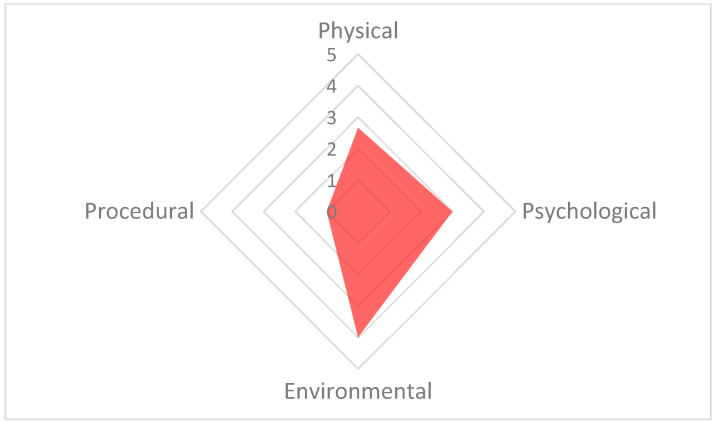
The welfare polygon for 13 September 15:30. The environmental parameter has the highest score (4), resulting from high scores for the factors: environment (5), group size and structure (6) and enclosure complexity (6). The psychological parameter is also high due to the factor of social interaction (5). The axes in this figure are adjusted to improve readability.

**Figure 7:**
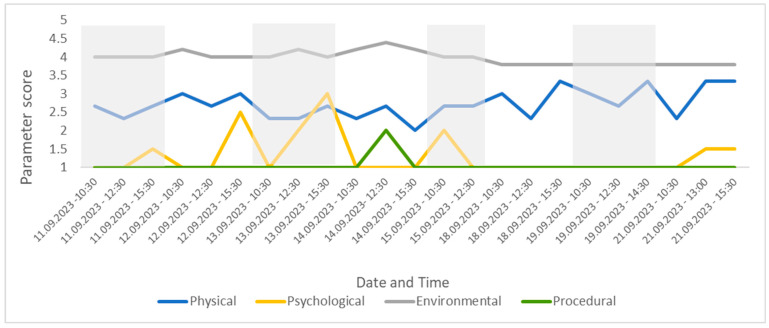
The four parameter scores for the group, presented over time for the study period. This figure shows changes in welfare related to specific parameters, allowing for the assessor to easily drill down into the CWAS.

**Figure 8:**
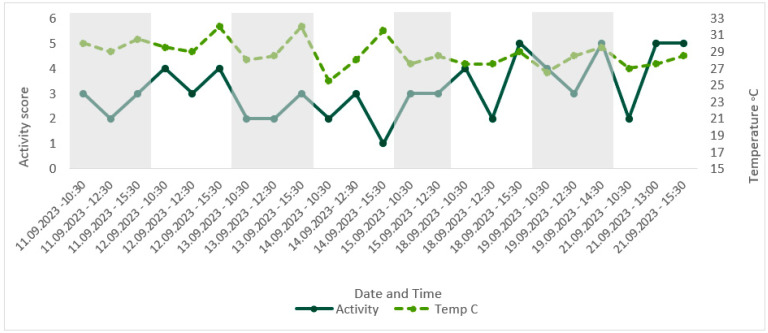
Comparison of score for the factor ‘activity’ over time, with corresponding enclosure temperature (°C). Activity during the day was deemed an indicator of poor welfare; therefore, an increase in score for activity represented an increase in group activity level.

Twenty-three observations were conducted across eight days. The average number of individuals seen per observation was 27 (max. 31, min. 21) due to leaflitter providing some cover but not enough for all the individuals present (the maximum out of sight during an assessment was 10; the average number out of sight was 4). Changes were detected in welfare score over this period and are visually presented in Figure 4, Figure 5, Figure 6, Figure 7 and Figure 8. Figure 4 depicts the change in the group’s CWAS over time. The maximum score that can be attained for a single assessment is 200. The maximum CWAS score for the group during the study was 13.3, whilst the minimum was 6.1 (Figure 4).

The poorest welfare score for the group (13.3) occurred on 13 September at the 15:30 assessment, whilst the best welfare score for the group (6.1) occurred on 21 September at the 10:30 assessment. The average welfare score for the group for the study period was 7.9 (Figure 5). The psychological parameter had the greatest impact on the CWAS for the 13 September 15:30 (Figure 6 and Table 5). The factors of environment, group size and structure, and enclosure complexity did not decline below scores of 5, 6 and 5, respectively, across the study period.

Changes to the four parameter scores can also be viewed across time. The psychological parameter exhibits the greatest change in score (min. 1–max. 3). The environmental parameter remains fairly consistent throughout the study and the procedural parameter deviates from 1 only once (14 September 23 12:30), due to the removal of old food by a keeper during the observation (Figure 7).

Temperatures remained above the minimum of 21–24 °C, as recommended in the husbandry-related literature throughout the study period, with the lowest recorded temperature of 25.5 °C [41,53]. The maximum temperature recorded was 32 °C, exceeding the maximum recommended temperature of 27–30 °C [41,53]. CWAS visually followed a similar trend to time of day (Figure 4). A Spearman’s rank correlation was computed using R [54] to assess the relationship between CWAS and time of day and found a weak but insignificant positive correlation, rs(21) = 0.36, *p* = 0.094. Activity level appeared to follow the same trend as temperature (Figure 8), and a Spearman’s rank correlation [54] found a very weak insignificant positive correlation between the two variables, rs(21) = 0.11, *p* = 0.607.

## 4. Discussion

The aim of this study was to expand the work by Narshi et al. [25] to examine whether the previously validated Animal Welfare Assessment Grid (AWAG) could be modified to objectively assess the welfare of a large group of terrestrial invertebrates. Through adapting the welfare scoring criteria for *Gromphadorhina oblongonota* at the group level and successfully trialling it with a group of 31 males at Marwell Zoo, we provide evidence the AWAG can be utilised to monitor the welfare of terrestrial invertebrates at the group level. With the recent passing of the Animal Welfare (Sentience) Bill into UK legislation in 2022, and the resulting questions and concerns for invertebrate welfare that this raised, in addition to the growing interest in farming invertebrates for human consumption, this is a timely result.

Although welfare is a subjective experience, assessing welfare at the group level leaves individual differences and individual personality unaccounted for. Group-level welfare assessments tend to focus more on what the animal has been provisioned with, harking back to the ‘Five Freedoms’, rather than assessing animal-based measures such as individual behaviour and physical and psychological health. However, through using the AWAG model, we included animal-based measures in this group-level welfare assessment and our results indicate that, through using this tool, changes in welfare and welfare trends over time can be identified and tracked at the group level. The results also show that it is possible to identify which factor(s) may be impacting welfare, allowing for animal care givers to focus on improving these. For *G. oblongonota,* our results suggest the environment, group size and structure, and enclosure complexity may impact baseline welfare score. In addition, the factors ‘Activity level’ under the physical parameter and ‘Social interaction’ under the psychological parameter varied the most between observations, and thus had the greatest impact on changes in welfare score over the course of this study.

### 4.1. The Impact of Environmental Factors on Welfare Score

As they are ectotherms, low temperatures can have a negative impact on the welfare of cockroaches. Low temperatures, i.e., 8–10 °C, can induce a ‘chill-coma’, where cockroaches lose mobility but can survive if temperatures are reversed before a chill-injury occurs [55]; therefore, it is important to monitor enclosure temperature closely. Although maximum temperatures of 27–30 °C are recommended in the literature [41,53], the temperature in *G. oblongonota’s* wild habitat in southern Madagascar can reach 40 °C. Except for increases in reproductive activity [41,53,55] and increased metabolic rate [56], we found no evidence in the literature for a negative impact of higher temperatures on welfare. Even so, we increased the welfare score when temperatures were above the recommended level in case, over time, correlations were found with other factors.

**Figure 9:**
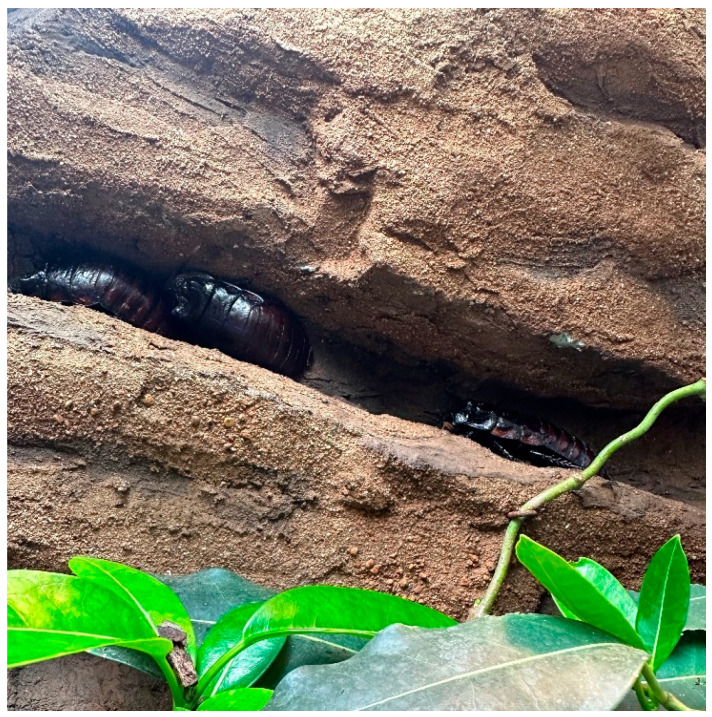
Three *Gromphadorhina oblongonota* exhibiting thigmotaxic and negative phototactic behaviour in one of the wall crevices of the enclosure. (Kilroy, D).

**Figure 10:**
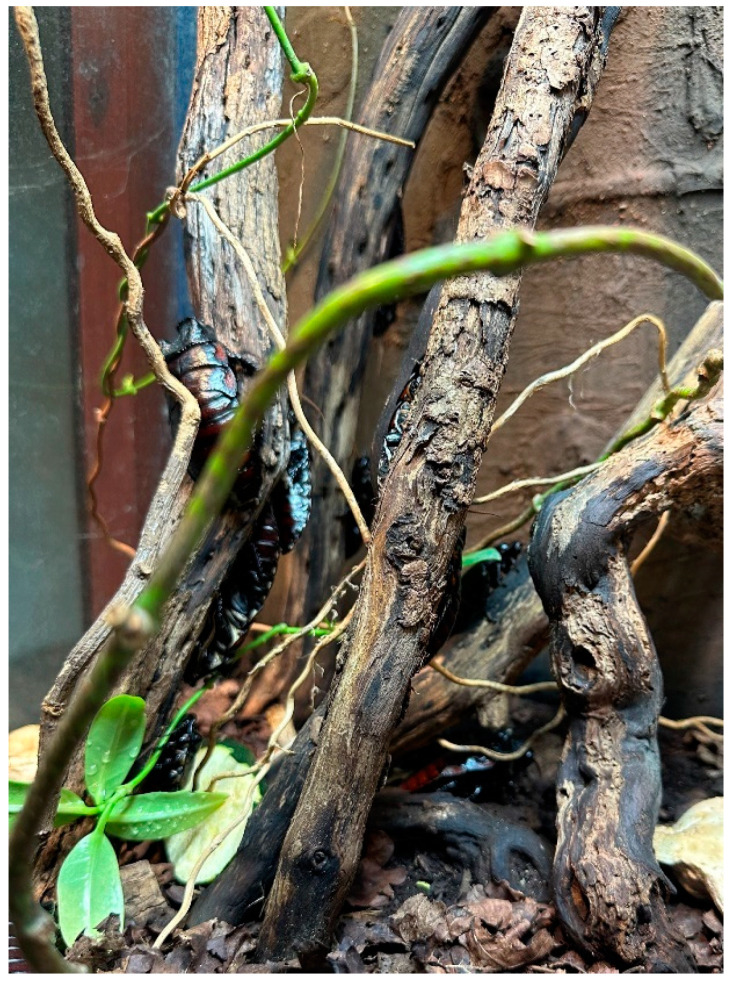
*Gromphadorhina oblongonota* in the enclosure at Marwell Zoo. Note the position of all bar one individual on the underside of the branches. (Kilroy, D).

The majority of cockroach species are negatively phototactic, meaning that they will move away from light. In this enclosure, there were very few options to move away from or seek shelter from the light, which was on a seasonal 13:11–11:13 cycle. Leaflitter provided some cover but not enough for all the individuals present. The remainder appeared to choose locations in the enclosure with as much cover as possible, either in the shallow cervices on the rock facades (Figure 9) or on the underside of the branches (Figure 10).

We noted anecdotally that when leaflitter was introduced, activity levels increased and many of the cockroaches moved to burrow into the new substrate. For negatively phototactic species, being unable to escape light can lead to increased stress and anxiety [57,58,59], negatively impacting welfare and resulting in lower survival rates [42].

Cockroaches are also positively thigmotaxic; that is, they will actively seek contact with objects in their environment. This behaviour increases safety and security, particularly from predators, and is often used as an escape response. Cockroaches tend to avoid open spaces and will preferentially locate themselves close to or touching walls or other vertical surfaces (Figure 9). It is likely that this behaviour is also associated with light avoidance [42]. Studies have shown that thigmotactic deprivation, e.g., lack of access to shelters, can lead to stress, resulting in reduced growth, increased energy consumption, reduced fecundity and reduced survival rate [42,60,61].

### 4.2. Other Factors That Impacted Welfare Score

We considered activity during the day to be a sign of compromised welfare due to the disruption of the circadian rhythm. Without comparison with other colonies or evidence for wild individuals, it is unknown whether the amount of activity seen during the day was typical for the species. By highlighting this using the AWAG, however, we were able to track changes over time. As ectotherms, temperature plays a key role in the activity level of *G. oblongonota*, with temperatures below 21 °C leading to reduced activity level and lethargy and high temperatures increasing activity level [41,53,55]. Activity level may therefore be an indicator for the impact of environmental parameters on welfare in this species.

Alongside ‘Group size and structure’, we identified ‘Social interaction’ as a key welfare indicator because it has been demonstrated previously that male *Gromphadorhina* exhibit extensive dominance hierarchies [47,49,50,62]. Although cockroaches are typically highly social and aggregate in large numbers, wild *Gromphadorhina* spp. usually live in mixed-sex groups of only ca. 10 individuals, in which males exhibit territoriality, combat behaviours, and dominance hierarchies [35,63]. These social dynamics suggest that high numbers of males in a small area will lead to an increase in these negative social interactions. As this group of 31 males was maintained at a fairly high stocking density, we used the frequency, severity, and number of individuals involved in negative interactions as a welfare indicator [38]. Although negative social interactions remained low in number and severity, and there were only two assessments in which we scored welfare a 4 (12 September 15:30) or 5 (13 September 15:30; Figure 6), it is possible that a stable social hierarchy had already been established prior to the start of the study [47] and that this factor will change more when there are changes to the group (e.g., new individuals are added to the enclosure). We observed some individuals to be missing parts of their antennae and tarsi, which could be a result of previous bouts of aggression or, alternatively, old age [39].

We did not observe any cockroach responses to guests. A lack of responses could have been a result of the assessors’ presence changing guest behaviour and leading to fewer negative behaviours such as banging on the glass window or shouting. Fewer responses could also have been because the enclosure was situated behind a glass window that lessened sound and vibrations. Alternatively, stationary behaviour may have continued because there were few hiding places for cockroaches, meaning escaping out of the sight of guests was not possible. Another explanation may be that presence if guests did not negatively impact welfare; however, this seems unlikely considering that the negative impact of guests on animal welfare has been described in detail (reviewed in Sherwen and Hemsworth [64]). Further research is required to determine whether there are more relevant methods for scoring the impact of guests on the welfare of invertebrates.

In addition to the results from this study supporting use of the AWAG for welfare assessments of groups, they will also feed directly into husbandry and enclosure changes for this group of *G. oblongonota*. These changes will include a larger enclosure, focussing on floor space rather than height as *G. oblongonota* are primarily terrestrial [36,65,66]. A greater floor area will also allow for gradients in temperature, humidity and light, providing choice and control over how the environment is used [1,39,53]. Changes will also include the introduction of canopy cover to reduce the amount of light falling on the floor, as would occur in the wild forest habitat, and the addition of more leaflitter and other shelters that can provide escape from the light and areas of higher humidity.

### 4.3. Limitations

Whilst we are satisfied that this trial was successful at modifying a welfare assessment tool for terrestrial invertebrates at the group level, the number of welfare assessments employed in this study was low. A long-term (≥95 days, as per Justice et al. [29]) evaluation of this modified AWAG scoring template to allow for an improved analysis of welfare trends over time is the next step. Adding nocturnal observations to the methods will allow for us to gain a better understanding of behaviour and whether the results obtained from diurnal observations only are a true representation of welfare. Unless organisations maintain their cockroach colonies in reverse light–dark cycle, a lack of nocturnal observations is likely to be a widespread issue. Once periods of peak activity are identified, observations can occur at those times to reduce the impact of time of day on the results. Another issue we faced was an inability to see all the individuals in the group during each assessment without causing a disturbance. Ideally, the assessment score would represent the total sum of the welfare of all the individuals in the group; however, in practice this can be difficult, so we are presenting a tool that we believe can still positively contribute to our understanding of group-level welfare within the confines of these limitations.

Abnormal behaviour is a potentially important welfare indicator [67,68,69]. We ended up not scoring it in this study because it was not possible to view the cockroaches at night, when they are most active and thus most likely to exhibit abnormal behaviour. It was also not possible to assess the frequency of hissing due to the enclosure being situated behind an additional glass window, meaning that hisses were not audible to the observers. This is something to address in future studies.

In this study, we modified the AWAG template for *G. oblongonota*. More research is necessary to determine whether a single generalised template could be designed for the assessment of multiple species of terrestrial invertebrates at the group level, taking into account differences in husbandry methods and environment. In addition, we based most factors in this modified AWAG on the percentage of individuals that deviated from the norm. Further research should explore combining percentages with severity (as per the factor ‘Social interaction’) to improve how representative the scores are for the welfare of the individual within the group. Furthermore, for the parameter averages to be truly representative, scores between 1 and 10 should uniformly increase or decrease. This should be accounted for in future studies.

One of the key steps in designing a welfare assessment tool is reviewing the current literature on animal welfare research and the species or taxa of concern. There are few studies of *G. oblongonota* welfare in captivity, resulting in a heavy reliance on information gathered from the wild concerning environment and behaviour. These data may not always be directly related to their welfare in captivity [70,71]. As a result, it is important to be conscious of the relevance of this information for the captive environment. In some situations, it may be preferable for welfare assessments to not compare captivity to the natural environment and instead concentrate on how well the environment fulfils the behavioural requirements of the species, giving greater weight to animal-based factors [33].

## 5. Conclusions

This study advances the welfare assessment of large groups of terrestrial invertebrates by adapting the Animal Welfare Assessment Grid (AWAG) for *Gromphadorhina oblongonota*. Our findings underscore its suitability as a practical, easy-to-use tool for objective group-level welfare evaluation. These are essential requirements for a monitoring tool, given that current individual welfare monitoring systems can be time-consuming and impractical for large groups [23,24]. These findings contribute to tangible improvements in the care of *G. oblongonota* in captivity, ultimately elevating their welfare standards but also contributing to the ability to monitor and improve welfare for groups of other invertebrate species.

Based on our findings, we would suggest the following recommendations for others looking to adapt the AWAG for use with terrestrial invertebrates:

- Adapt the AWAG scoring criteria to the species, based on thorough research.
- Improve the representation of individual welfare by combining the percentage of impacted individuals and severity of the impact in the scoring criteria.
- Standardise the time of day the assessment takes place, which should include the species’ most active period.
- Assess welfare regularly.
- Utilise the assessment results to drive changes that will improve animal welfare.

## Figures and Tables

**Table 1:** Scores for assessing factors within the physical parameter, adapted from Narshi et al. [25].

Score	General Condition	Presence of Injury	Activity Level
	Reduction in general condition would include: dull carapace, difficulty moulting, abnormal body morphology (abdomen not shrivelled/swollen), high density of mites.	Presence of injury including: damaged or missing limbs, tarsi or antennae, lameness/abnormal locomotion, prolapse.	Activity level, e.g., lethargy or hyperactivity. Consider circadian rhythm, which is normally high at night.
1	All of group demonstrated ideal physical condition.	No observable signs of injury.	All of the group demonstrated normal activity levels.
2	1–10% had worse than optimum physical condition.	1–10% had observable signs of injury.	1–10% did not exhibit normal activity levels.
3	11–20% had worse than optimum physical condition.	11–20% had observable signs of injury.	11–20% did not exhibit normal activity levels.
4	21–30% had worse than optimum physical condition.	21–30% had observable signs of injury.	21–30% did not exhibit normal activity levels.
5	31–40% had worse than optimum physical condition.	31–40% had observable signs of injury.	31–40% did not exhibit normal activity levels.
6	41–50% had worse than optimum physical condition.	41–50% had observable signs of injury.	41–50% did not exhibit normal activity levels.
7	51–60% had worse than optimum physical condition.	51–60% had observable signs of injury.	51–60% did not exhibit normal activity levels.
8	61–70% had worse than optimum physical condition.	61–70% had observable signs of injury.	61–70% did not exhibit normal activity levels.
9	71–80% had worse than optimum physical condition.	71–80% had observable signs of injury.	71–80% did not exhibit normal activity levels.
10	>80% had worse than optimum physical condition.	>80% had observable signs of injury.	>80% did not exhibit normal activity levels.

**Table 2:** Scores for assessing factors within the psychological parameter, adapted from Narshi et al. [25].

Score	Abnormal Behaviour	Response to Guest Presence	Social Interaction
	Abnormal behaviour such as moving fast and excessive hissing. Remember to consider normal circadian rhythm when making the assessment.	Reaction to people approaching glass or sudden elevations in noise level. Reactions include: increased startling and hissing.	Evidence of aggression (threat displays or combative) or defensive/submissive behaviours or, e.g., abdominal flick, push, butt or lunge, abdominal extension, abdominal thrash, agonistic hiss and stilt stance [47].
1	No abnormal behaviours observed in any of the group.	None of the group reacted to guest presence.	No negative social interactions witnessed, either aggressive or defensive.
2	1–10% exhibited abnormal behaviours.	1–10% reacted to guest presence.	1 mild incident witnessed.
3	11–20% exhibited abnormal behaviours.	11–20% reacted to guest presence.	Multiple mild incidences between 2 individuals.
4	21–30% exhibited abnormal behaviours.	21–30% reacted to guest presence.	1 moderate incidence witnessed.
5	31–40% exhibited abnormal behaviours.	31–40% reacted to guest presence.	Multiple mild incidences between >2 individuals.
6	41–50% exhibited abnormal behaviours.	41–50% reacted to guest presence.	Multiple moderate incidences between 2 individuals
7	51–60% exhibited abnormal behaviours.	51–60% reacted to guest presence.	1 severe incidence witnessed
8	61–70% exhibited abnormal behaviours.	61–70% reacted to guest presence.	Multiple severe incidences between 2 individuals
9	71–80% exhibited abnormal behaviours.	71–80% reacted to guest presence.	>80% of individuals involved in at least 1 mild incident.
10	>80% or more of the group exhibited abnormal behaviours.	>80% reacted to guest presence.	>50% of individuals involved in at least 1 moderate-severe incident.

**Table 3:** Scores for assessing factors within the environmental parameter, adapted from Narshi et al. [25].

Score	Environment	Group Size and Structure	Enclosure Complexity	Nutrition	Contingent Events
	Suitability of the environment for the species, e.g., location in the zoo, guest viewing, temperature (22–28 °C plus gradient), humidity (60–80%), space, lighting and light cycle, ventilation (no drafts), clean (no mould), drainage, low noise levels.	Considering the number of individuals, group structure, and density in the enclosure.	The enclosure simulates the natural habitat, including branches, bark, leaf litter, rock crevices, among other dark, damp locations, with various heights. Suitable substrate is offered, such as organic soil and leaf litter and natural forage options.	Refers to diet and forage, and presentation.	Contingent events include: enclosure changes, building works, guest events/educational aids, bin collection, deliveries.
1	The enclosure is ideal for the species.	Group size in accordance with natural group size; group structure is appropriate; suitable density for the enclosure size.	Ability to demonstrate all natural behaviours.	Nutrition is optimally suited to the species and individual. (nutritional, physiological and behavioural).	None.
2	One factor is below average.	Group structure is marginally different from appropriate group structure.	The ability to demonstrate natural behaviours, but the available options for this are not ideal.	Nutrition available has a marginally decreased appropriateness to accommodate species-specific needs.	Event outside of the enclosure (e.g., continuing construction work) taking place with little disruption
3	Two/three factors are below average.	An increased or decreased number of animals present in comparison to the natural group size range, no overstocking.	The ability to demonstrate natural behaviours, but there are few possibilities for this.	Nutrition available has a moderately decreased appropriateness for species-specific needs.	Event outside of the enclosure (e.g., continuing construction work) with slight disruption, e.g., noise or vibrations
4	Four/five factors are below average.	Group structure is moderately different from appropriate group structure.	The ability to demonstrate one form of natural behaviour is constrained due to the enclosure design.	Nutrition available is largely inappropriate to accommodate species-specific needs.	An external event that causes some disruption OR a change in the enclosure’s contents without any other events occurring.
5	Six factors below average.	A somewhat greater animal density than suitable for enclosure size (many young present without a decrease in the adult population)	A certain type of natural behaviour is unable to be demonstrated because the option is not offered.	Nutrition available is inadequate to fulfil behavioural needs of the species.	External incident that causes a visible interruption OR movement into a familiar environment without any other events occurring.
6	Seven factors below average.	An increased or decreased number of animals present in comparison to the natural group size range, with marginal overstocking.	The options to demonstrate natural behaviours are limited, preventing the demonstration of particular natural behaviours associated with enclosure design.	Nutrition available is inadequate to fulfil physiological needs of the species.	External incident that causes a visible interruption AND movement into a familiar environment.
7	Eight factors below average.	Group structure is greatly different from appropriate group structure.	The options to demonstrate natural behaviours are limited, preventing the demonstration of multiple natural behaviours associated with enclosure design.	Nutrition available is inadequate to fulfil the behavioural and physiological needs of the species.	Movement into unfamiliar enclosure OR addition of unfamiliar animal.
8	Nine factors below average.	Animal density is moderately greater than suitable for enclosure size.	The options to demonstrate natural behaviours are limited, preventing the demonstration of the majority of natural behaviours associated with enclosure design.	Available nutrition is inadequate to fulfil behavioural, physiological and nutritional needs of the species.	Movement into unfamiliar enclosure AND addition of unfamiliar animal.
9	Ten factors below average.	Animal density is much greater than enclosure can accommodate.	The options to demonstrate natural behaviours are very limited, preventing the demonstration of virtually all of the natural behaviours associated with enclosure design.	No nutrition is available.	External incident that causes a definite interruption AND movement into unfamiliar enclosure
10	All factors scored are inadequate—the enclosure is inappropriate for the species being monitored.	Group size greatly differed from natural group size or a significant level of overstocking.	The animal is unable to demonstrate natural behaviours associated with enclosure design because the options are not offered.	Nutrition is dangerous for the species.	Mixture of events: extended external incident, movement into an unfamiliar enclosure, introduction of unfamiliar animals. Extreme detrimental levels of disruption.

**Table 4:** Scores for assessing factors within the procedural parameter, adapted from Narshi et al. [25].

Score	Effect of Intervention
	For example, removing old food or dead individuals, changes to environmental complexity, etc. Stress-related behaviours include: hissing, burrowing or fast movement away from the disturbance
1	No intervention.
2	1–10% reacted to the intervention by exhibiting stress-related behaviours.
3	11–20% reacted to the intervention by exhibiting stress-related behaviours.
4	21–30% reacted to the intervention by exhibiting stress-related behaviours.
5	31–40 reacted to the intervention by exhibiting stress-related behaviours.
6	41–50% reacted to the intervention by exhibiting stress-related behaviours.
7	51–60% reacted to the intervention by exhibiting stress-related behaviours.
8	61–70% reacted to the intervention by exhibiting stress-related behaviours.
9	71–80% reacted to the intervention by exhibiting stress-related behaviours.
10	>80% reacted to the intervention by exhibiting stress-related behaviours.

**Table 5:** Factor scores for 13 September 15:30. Highlighted in red are the three factors that resulted in the environmental parameter having the highest score for that assessment and the single factor that raised the psychological parameter.

Parameter/Factor	Score	Parameter/Factor	Score
**Physical**		**Environmental**	
General condition	2	Environment	5
Presence of injury	3	Group size and structure	6
Activity level	3	Enclosure complexity	6
**Psychological**		Nutrition	2
Response to guests	1	Contingent events	1
Social interaction	5	**Procedural**	
		Effect of intervention	1

## Data Availability

The data presented in this study are available on request from the corresponding author.

## References

[1] Cooper J.E. (2004). Invertebrate Care. Vet. Clin. Exot. Anim. Pract..

[2] Boppré M., Vane-Wright R.I., Carere C., Mather J., Animal Welfare (2019). Welfare Dilemmas Created by Keeping Insects in Captivity. The Welfare of Invertebrate Animals.

[3] van Huis A. (2019). Welfare of Farmed Insects. J. Insects Food Feed..

[4] Delvendahl N., Rumpold B.A., Langen N. (2022). Edible Insects as Food–Insect Welfare and Ethical Aspects from a Consumer Perspective. Insects.

[5] Pippinato L., Gasco L., Di Vita G., Mancuso T. (2020). Current Scenario in the European Edible-Insect Industry: A Preliminary Study. J. Insects Food Feed..

[6] WWF-UK (2022). The Future of Feed: A WWF Roadmap to Accelerating Insect Protein in UK Feeds. https://www.wwf.org.uk/sites/default/files/2022-06/future_of_feed_full_report.pdf.

[7] The Member States (2007). Treaty of Lisbon Amending the Treaty on European Union and the Treaty Establishing the European Community, Signed at Lisbon, 13 December 2007.

[8] Birch J., Burn C., Schnell A., Browning H., Crump A. (2021). Review of the Evidence of Sentience in Cephalopod Molluscs and Decapod Crustaceans.

[9] Jinman P. Animal Sentience 2018. https://assets.publishing.service.gov.uk/media/5f0f05db3a6f400394d55225/FAWC_letter_on_animal_sentience_16_March_2018.pdf.

[10] Lambert H., Elwin A., D’Cruze N. (2021). Wouldn’t Hurt a Fly? A Review of Insect Cognition and Sentience in Relation to Their Use as Food and Feed. Appl. Anim. Behav. Sci..

[11] Sherwin C.M. (2001). Can Invertebrates Suffer? Or, How Robust Is Argument-By-Analogy?. Anim. Welf..

[12] Knutsson S., Munthe C. (2017). A Virtue of Precaution Regarding the Moral Status of Animals with Uncertain Sentience. J. Agric. Environ. Ethics.

[13] Barrett M., Chia S.Y., Fischer B., Tomberlin J. (2022). Welfare Considerations for Farming Black Soldier Flies, Hermetia Illucens (Diptera: Stratiomyidae): A Model for the Insects as Food and Feed Industry. J. Insects Food Feed..

[14] Saul-Gershenz L.S., Arnold R.A., Scriber J.M., Gibbons E.F., Durrant B.S., Demarest J. (1995). Design of Captive Environments for Endangered Invertebrates. Conservation of Endangered Species in Captivity: An Interdisciplinary Approach.

[15] Arbuckle K. (2013). Folklore Husbandry and a Philosophical Model for the Design of Captive Management Regimes. Herpetol. Rev..

[16] WAZA (2023). Our Approach to Animal Welfare. https://www.waza.org/.

[17] Arndt S.S., Goerlich V.C., van der Staay F.J. (2022). A Dynamic Concept of Animal Welfare: The Role of Appetitive and Adverse Internal and External Factors and the Animal’s Ability to Adapt to Them. Front. Anim. Sci..

[18] Duncan I.J.H. (2006). The Changing Concept of Animal Sentience. Appl. Anim. Behav. Sci..

[19] Duncan I.J.H. (2016). Is Sentience Only a Nonessential Component of Animal Welfare?. Anim. Sentience.

[20] Cooper J.E., Williams D.L. (2014). The Feeding of Live Food to Exotic Pets: Issues of Welfare and Ethics. J. Exot. Pet Med..

[21] Keller M. (2017). Feeding Live Invertebrate Prey in Zoos and Aquaria: Are There Welfare Concerns?. Zoo Biol..

[22] Kagan R., Allard S., Carter S. (2018). What Is the Future for Zoos and Aquariums?. J. Appl. Anim. Welf. Sci..

[23] Collins S., Burn C.C., Wathes C.M., Cardwell J.M., Chang Y.-M., Bell N.J. (2021). Time-Consuming, but Necessary: A Wide Range of Measures Should Be Included in Welfare Assessments for Dairy Herds. Front. Anim. Sci..

[24] DiVincenti L., McDowell A., Herrelko E.S. (2023). Integrating Individual Animal and Population Welfare in Zoos and Aquariums. Animals.

[25] Narshi T.M., Free D., Justice W.S.M., Smith S.J., Wolfensohn S. (2022). Welfare Assessment of Invertebrates: Adapting the Animal Welfare Assessment Grid (AWAG) for Zoo Decapods and Cephalopods. Animals.

[26] Reuben Digital Animal Welfare Assessment Grid. https://awag.org.uk/.

[27] Mellor D.J., Beausoleil N.J., Littlewood K.E., McLean A.N., McGreevy P.D., Jones B., Wilkins C. (2020). The 2020 Five Domains Model: Including Human–Animal Interactions in Assessments of Animal Welfare. Animals.

[28] Honess P., Wolfensohn S. (2010). The Extended Welfare Assessment Grid: A Matrix for the Assessment of Welfare and Cumulative Suffering in Experimental Animals. Altern. Lab. Anim..

[29] Justice W.S.M., O’Brien M.F., Szyszka O., Shotton J., Gilmour J.E.M., Riordan P., Wolfensohn S. (2017). Adaptation of the Animal Welfare Assessment Grid (AWAG) for Monitoring Animal Welfare in Zoological Collections. Vet. Rec..

[30] Wolfensohn S., Shotton J., Bowley H., Davies S., Thompson S., Justice W. (2018). Assessment of Welfare in Zoo Animals: Towards Optimum Quality of Life. Animals.

[31] Ryan M., Waters R., Wolfensohn S. (2021). Assessment of the Welfare of Experimental Cattle and Pigs Using the Animal Welfare Assessment Grid. Animals.

[32] Malkani R., Paramasivam S., Wolfensohn S. (2022). Preliminary Validation of a Novel Tool to Assess Dog Welfare: The Animal Welfare Assessment Grid. Front. Vet. Sci..

[33] Free D., Justice W.S.M., Smith S.J., Howard V., Wolfensohn S. (2022). An Approach to Assessing Zoo Animal Welfare in a Rarely Studied Species, the Common Cusimanse *Crossarchus obscurus*. J. Zool. Bot. Gard..

[34] Nelson M.C., Fraser J. (1980). Sound Production in the Cockroach, *Gromphadorhina portentosa*: Evidence for Communication by Hissing. Behav. Ecol. Sociobiol..

[35] Schal C., Gautier J.-Y., Bell W.J. (1984). Behavioural Ecology of Cockroaches. Biol. Rev..

[36] Monahan C.F., Bogan J.E., LaDouceur E.E.B. (2023). Histological Findings in Captive Madagascar Hissing Cockroaches (*Gromphadorhina portentosa*) and a Literature Review. Vet. Pathol..

[37] Bell W.J., Roth L.M., Nalepa C.A. (2007). Cockroaches: Ecology, Behavior, and Natural History.

[38] Brereton J., Rose P. (2022). The Behavioural Biology of Invertebrates. The Behavioural Biology of Zoo Animals.

[39] Kandilian K. (2022). Roach Crossing’s Cockroach Husbandry Guide.

[40] Yoder J.A., Barcelona J.C. (1995). Food and Water Resources Used by the Madagascan Hissing-Cockroach Mite, Gromphadorholaelaps Schaeferi. Exp. Appl. Acarol..

[41] Chua J., Fisher N.A., Falcinelli S.D., DeShazer D., Friedlander A.M. (2017). The Madagascar Hissing Cockroach as an Alternative Non-Mammalian Animal Model to Investigate Virulence, Pathogenesis, and Drug Efficacy. J. Vis. Exp..

[42] Retardo-Agua J., Crausos K., Sasam J.M. (2023). Growth Rate and Thigmotactic Behavior of Turkestan Cockroach (*Blatta lateralis*) under Different Illumination Conditions. IJRIAS.

[43] Michel M., Lyons L.C. (2014). Unraveling the Complexities of Circadian and Sleep Interactions with Memory Formation through Invertebrate Research. Front. Syst. Neurosci..

[44] Stephenson R., Chu K.M., Lee J. (2007). Prolonged Deprivation of Sleep-like Rest Raises Metabolic Rate in the Pacific Beetle Cockroach, *Diploptera punctata* (Eschscholtz). J. Exp. Biol..

[45] Mishra M., Meyer-Rochow V.B. (2008). Fine structural description of the compound eye of the Madagascar ‘hissing cockroach’*Gromphadorhina portentosa* (Dictyoptera: Blaberidae). Insect Sci..

[46] Warrant E.J. (2017). The remarkable visual capacities of nocturnal insects: Vision at the limits with small eyes and tiny brains. Philos. Trans. R. Soc. B Biol. Sci..

[47] Clark D.C., Moore A.J. (1994). Social Interactions and Aggression among Male Madagascar Hissing Cockroaches (*Gromphadorhina portentosa*) in Groups (Dictyoptera: Blaberidae). J. Insect Behav..

[48] Clark D.C., Moore A.J. (1995). Variation and Repeatability of Male Agonistic Hiss Characteristics and Their Relationship to Social Rank in *Gromphadorhina portentosa*. Anim. Behav..

[49] Clark D., Moore A. (1995). Social Communication in the Madagascar Hissing Cockroach: Features of Male Courtship Hisses and a Comparison of Courtship and Agonistic Hisses. Behaviour.

[50] Mack C. (2022). Winner and Loser Effects in Madagascar Hissing Cockroaches (Gromphadorhina portentosa).

[51] Brouwers S., Duchateau M.J. (2021). Feasibility and Validity of the Animal Welfare Assessment Grid to Monitor the Welfare of Zoo-Housed Gorillas Gorilla Gorilla Gorilla. J. Zoo Aquar. Res..

[52] Analog Devices Inc. (2013). Thermochron iButton.

[53] Mulder P., Shufran A. (2016). Madagascar Hissing Cockroaches: Information and Care.

[54] R Core Team (2020). R: A Language and Environment for Statistical Computing.

[55] Bradt D.L., Hoback W.W., Kard B.M. (2018). American Cockroach Response to Cold Temperatures. Southwest. Entomol..

[56] Halsey L.G., Matthews P.G.D., Rezende E.L., Chauvaud L., Robson A.A. (2015). The interactions between temperature and activity levels in driving metabolic rate: Theory, with empirical validation from contrasting ectotherms. Oecologia.

[57] Arrant A.E., Schramm-Sapyta N.L., Kuhn C.M. (2013). Use of the Light/Dark Test for Anxiety in Adult and Adolescent Male Rats. Behav. Brain Res..

[58] Zhukovskaya M., Novikova E., Saari P., Frolov R.V. (2017). Behavioral Responses to Visual Overstimulation in the Cockroach *Periplaneta americana* L.. J. Comp. Physiol. A.

[59] Zewde A.M., Yu F., Nayak S., Tallarida C., Reitz A.B., Kirby L.G., Rawls S.M. (2018). PLDT (Planarian Light/Dark Test): An Invertebrate Assay to Quantify Defensive Responding and Study Anxiety-like Effects. J. Neurosci. Methods.

[60] Laurent Salazar M.-O., Planas-Sitjà I., Sempo G., Deneubourg J.-L. (2018). Individual Thigmotactic Preference Affects the Fleeing Behavior of the American Cockroach (Blattodea: Blattidae). J. Insect Sci..

[61] Chen Y.-R., Li D.-W., Wang H.-P., Lin S.-S., Yang E.-C. (2022). The Impact of Thigmotaxis Deprivation on the Development of the German Cockroach (*Blattella germanica*). iScience.

[62] Yoder K. (2014). Food Resource Based Agonistic Interactions in Madagascar Hissing Cockroaches (*Gromphadorhina portentosa*). https://www.researchgate.net/publication/269410065_Food_Resource_Based_Agonistic_Interactions_in_Madagascar_Hissing_Cockroaches_Gromphadorhina_portentosa#fullTextFileContent.

[63] Varadínová Z., Stejskal V., Frynta D. (2010). Patterns of Aggregation Behaviour in Six Species of Cockroach: Comparing Two Experimental Approaches: Aggregation Behaviour of Cockroaches. Entomol. Exp. Appl..

[64] Sherwen S.L., Hemsworth P.H. (2019). The Visitor Effect on Zoo Animals: Implications and Opportunities for Zoo Animal Welfare. Animals.

[65] van Casteren A., Codd J.R. (2010). Foot Morphology and Substrate Adhesion in the Madagascan Hissing Cockroach, *Gromphadorhina portentosa*. J. Insect Sci..

[66] Clark D., Shanklin D. (1995). Madagascar Hissing Cockroaches (Gromphadorhina portentosa).

[67] Broom D.M. (1986). Indicators of Poor Welfare. Br. Vet. J..

[68] Hill S.P., Broom D.M. (2009). Measuring Zoo Animal Welfare: Theory and Practice. Zoo Biol..

[69] Manteca X., Amat M., Salas M., Temple D. (2016). Animal-Based Indicators to Assess Welfare in Zoo Animals. CABI Rev..

[70] Veasey J.S., Waran N.K., Young R.J. (1996). On Comparing the Behaviour of Zoo Housed Animals with Wild Conspecifics as a Welfare Indicator. Anim. Welf..

[71] Howell C.P., Cheyne S.M. (2019). Complexities of Using Wild versus Captive Activity Budget Comparisons for Assessing Captive Primate Welfare. J. Appl. Anim. Welf. Sci..

